# Biomodification of PCL Scaffolds with Matrigel, HA, and SR1 Enhances *De Novo* Ectopic Bone Marrow Formation Induced by rhBMP-2

**DOI:** 10.1089/biores.2015.0020

**Published:** 2015-06-01

**Authors:** Wenjing Bao, Mei Gao, Yanyan Cheng, Hyun Jae Lee, Qinghao Zhang, Susan Hemingway, Zhibo Luo, Andrzej Krol, Guanlin Yang, Jing An

**Affiliations:** ^1^ Department of Pharmacology, SUNY Upstate Medical University, Syracuse, New York.; ^2^ Cancer Research Institute, SUNY Upstate Medical University, Syracuse, New York.; ^3^ Department of Medicine, Liaoning University of Traditional Chinese Medicine, Shenyang, China.; ^4^ Department of Radiology, SUNY Upstate Medical University, Syracuse, New York.

**Keywords:** *de novo* bone marrow formation, Matrigel, PCL scaffolds, rhBMP-2

## Abstract

The *de novo* formation of ectopic bone marrow was induced using 1.2-mm-thin polycaprolactone (PCL) scaffolds biomodified with several different biomaterials. *In vivo* investigations of *de novo* bone and bone marrow formation indicated that subcutaneous implantation of PCL scaffolds coated with recombinant human bone morphogenetic protein-2 (rhBMP-2) plus Matrigel, hydroxyapatite (HA), or StemRegenin 1 (SR1) improved formation of bone and hematopoietic bone marrow as determined by microcomputed tomography, and histological and hematopoietic characterizations. Our study provides evidence that thin PCL scaffolds biomodified with Matrigel, HA, and SR1 mimic the environments of real bone and bone marrow, thereby enhancing the *de novo* ectopic bone marrow formation induced by rhBMP-2. This ectopic bone marrow model will serve as a unique and essential tool for basic research and for clinical applications of postnatal tissue engineering and organ regeneration.

## Introduction

A complex dialogue occurs *in vivo* among the multiple lineages of cells operating within the dynamic three-dimensional (3D) bone marrow microenvironment and regulates the diverse cellular processes of hematopoiesis during both normal and abnormal development.^1–5^ However, the precise molecular and cellular mechanisms by which the stromal microenvironment regulates the fate of hematopoietic stem cells (HSCs) and their progenitor/precursor cells remain largely unexplored.^6–9^ This knowledge gap is primarily due to the lack of adequate models and technologies that can mimic and maintain a precise facsimile of the 3D microenvironment existing in real organs. Current developmental model systems, which include 2D and 3D *in vitro* tissue culture systems as well as transgenic and xenograft human tissue-*SCID* animals, are instructive, but they tend to be inadequate for human tissue reconstitutions due to the difficulty in manipulation or the lack of a real humanized 3D microenvironment.^10–14^ These challenges stress the importance of developing novel, appropriate, and manageable 3D models that resemble authentic bone marrow.

The present study arose from our previous work, where we demonstrated that decalcified bone matrix powder (DBMP) coupled with recombinant human bone morphogenetic protein-2 (rhBMP-2) induced the formation of a functional *de novo* hematopoietic microenvironment (HM).^15,16^ This *de novo* HM was capable of supporting a full spectrum of hematopoiesis in adult animals. However, the DBMP that we used is a very loose powder, which creates difficulties when attempting to control the shape and the thickness of the *de novo* generated ossicles. This disadvantage complicates the reculturing of the resulting ossicles and the maintenance of their *in vitro* stem cell and progenitor activities. We recently employed ready-made thin polycaprolactone (PCL) scaffolds^17,18^ and coupled these with our comprehensive combinations of rhBMP-2 plus Matrigel, hydroxyapatite (HA), and/or StemRegenin 1 (SR1). The result was the successful development of new manageable *de novo* generated bone marrow models with controllable thicknesses and shapes. This finding emphasizes the potential for biomodification of PCL scaffolds with Matrigel, HA, and SR1, for the enhancement of the *de novo* ectopic bone marrow formation induced by rhBMP-2.

## Materials and Methods

### Materials

Protein rhBMP-2 was purchased from Cell Guidance Systems. HA and 3D Biotek PCL scaffolds (5 mm in diameter, 1.2 mm high, and with a pore size of 569 μM) were purchased from Sigma-Aldrich, Inc. MethoCult™ GF M3434 and blunt-end needles were purchased from Stemcell Technologies, Inc. SR1 was purchased from the Cayman Chemical Company. The Matrigel matrix was purchased from BD Biosciences. The anti-Sca-1-FITC antibody was purchased from Miltenyi Biotec. The Alkaline Phosphatase (ALP) Assay Kit 50-489-198 was purchased from Bioassay Systems. The ATDC5 chondrogenic cell line was purchased from Sigma.

### Preparation of biomodified PCL scaffolds

Gelatin capsules containing biomodified PCL scaffolds were prepared before subcutaneous implantation. Experimental groups were divided by the type of coating on the PCL scaffold (Group 1 [negative/vehicle control]: phosphate-buffered saline [PBS]/0.1% bovine serum albumin [BSA]/0.1% dimethyl sulfoxide; Group 2: 10 μg rhBMP-2; Group 3: 10 μL Matrigel plus 10 μg rhBMP-2; Group 4: 2 mg HA,^19^ 10 μL Matrigel, and 10 μg rhBMP-2; Group 5: 2 mg HA, 10 μL Matrigel, 20 μg SR1, and 10 μg rhBMP-2; Group 6: 2 mg HA; Group 7: 10 μL Matrigel; Group 8: 20 μg SR1; Group 9: 2 mg HA and 10 μL Matrigel; and Group 10: 2 mg HA, 10 μL Matrigel, and 20 μg SR1). The whole procedure for biomodified scaffold preparation was performed on ice.

### Subcutaneous implantation of biomodified PCL scaffolds

Female mice (C57BL/6,∼22 g) aged 5–6 weeks were purchased from Charles River Laboratories International, Inc. One week after housing, gelatin capsules containing biomodified scaffolds were implanted subcutaneously into animals according to the methods previously published by our group.^15^ Briefly, mice were anesthetized by intraperitoneal injection of 100 mg/kg ketamine and 10 mg/kg xylazine. Under aseptic conditions, four or five capsules were implanted under the skin of the abdomen in each mouse from every group. At 8 weeks postimplantation, the mice were examined by microcomputed tomography (CT) examination or euthanized, and the scaffolds were harvested and processed for histological or hematopoietic analyses.

### Micro-CT measurement

Micro-CT imaging was performed using a MicroCATII scanner (Siemens) following published methods.^20,21^ Animals were anesthetized using a nonrebreathing anesthetic machine that delivers isoflurane/O_2_ anesthetic during *in vivo* scans to prevent motion artifacts. The anesthesia system consisted of an induction chamber and a scanning chamber. After the animal was placed and secured in the scanning chamber, the region of interest was positioned close to the central scanner axis. The caudal end was placed closest to the micro-CT gantry, with the rostral end held in place against an anesthesia delivery plastic cone (attached to the isoflurane anesthesia machine) that covered the tip of the animal's nose. The animal was somewhat extended and held in place with a tape to guarantee a correct anatomical position (i.e., straight spine). A scout scan was performed to select the CCD camera exposure time to reach 40–60% saturation, to verify correct alignment of the animal, and to allow proper positioning of the animal holder by centering the scan acquisition area at the region of interest. Thereafter, series of dark current and blank-field (background) images were acquired. The micro-CT scan was then performed (120 projection views, 8-sec exposure time per view, with 80 kVp and 0.5 mA technique, 1-mm Al filter). The tomographic reconstruction was performed using EXXIM software with 63 μm^3^ cubic voxels. The spatial resolution of the reconstructed images was about 120 μm. For each ossicle specimen, six fields per subregion per quadrant were randomly selected for density analysis with ImageJ, 1.48 (NIH).

### Histological staining of *de novo* generated ossicles

Ossicle samples were fixed in 10% buffered neutral formalin for up to 5 days, then decalcified as described previously,^22,23^ and embedded in paraffin. Three paraffin sections from each group were stained with hematoxylin and eosin (H&E) using routine protocols and another three sections were trichrome stained using an HT15 kit (Sigma).

### Hematopoietic colony-forming activity assay

Eight weeks after implantation, the femoral shaft and *de novo* generated ossicle samples were removed from every euthanized animal for preparation of cell suspensions. All samples were flushed several times with cold regular Iscove's Modified Dulbecco's Medium (IMDM; supplemented with 10% fetal bovine serum [FBS], 2 mM l-glutamine, 100 U/mL penicillin, and 100 μg/mL streptomycin). The samples were incubated on ice in the RBC lysis buffer containing 0.8% NH_4_Cl and 0.1 mM ethylenediaminetetraacetic acid (EDTA; pH=7.4) for 10 min and the resulting cell suspensions were then centrifuged at 300 *g* for 10 min. After washing the cells twice with IMDM and centrifugation, a colony-forming-unit (CFU) assay was performed according to the protocols published elsewhere.^15^ Cells were plated in MethoCult GF M3434 at a final concentration of 5×10^4^ cells/mL per 35-mm dish and incubated at 37°C, 5% CO_2_, and 95% humidity for 12 days. The numbers of CFUs were scored using a Nikon TE2000-U microscope equipped with a digital camera.

### Flow cytometry

The HSCs and progenitor cells from authentic femoral and ectopic ossicle marrow (OM) were quantified using the anti-Sca-1-FITC antibody and a flow cytometer. The cells were collected by flushing the femur and OM with regular IMDM using 25G needles. After gently pipetting the cell suspensions several times, the cells underwent two washes with the MACS buffer (PBS/0.5% BSA/2 mM EDTA) at 1 mL per 10^7^ total cells and centrifugation at 300 *g* for 10 min each time. The cells were then incubated with the anti-Sca-1-FITC antibody (1:10) at 4°C for 10 min. The cells were again washed twice with the MACS buffer and then analyzed by flow cytometry (BD LSR II).

### ALP activity

The ATDC5 chondrogenic cells (99072806-1VL) were cultured at 37°C in Dulbecco's Modified Eagle's Medium containing 10% FBS, 2 mM l-glutamine, 100 U/mL penicillin, and 100 μg/mL streptomycin. The ALP activity was assessed 3 days after treatment of subconfluent ADTC5 cells with 0 or 0.25 μM SR1 in the presence or absence of 10 ng/mL rhBMP-2. The ALP activity was measured with an ALP detection kit (50-489-198) and recording the absorbance at 405 nm with a microplate reader (Synergy II; Bioteck). Experiments were conducted in triplicate, and data were normalized against control cells treated with vehicle alone.

### Statistical analysis

Statistical analysis was performed using the one-way analysis of variance, followed by Tukey's Multiple Comparison Test (Prism 5.0 for Windows; GraphPad Software). Average values are expressed as mean±SE, *n*≥3. A *p*-value less than 0.05 was considered statistically significant.

## Results

### *De novo* formation of ectopic ossicles induced by biomodified PCL scaffolds

**FIG. 1.**
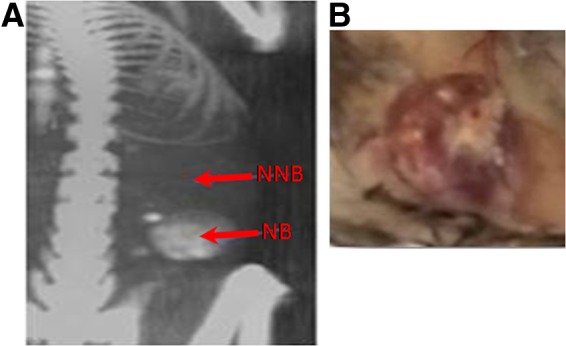
Representative three-dimensional microcomputed tomography (CT) image and morphology of *de novo* ossicles. **(A)** Micro-CT image of a mineralized ossicle at 8 weeks after implantation of polycaprolactone (PCL) scaffolds coupled with recombinant human bone morphogenetic protein-2 (rhBMP-2), hydroxyapatite (HA), Matrigel, and StemRegenin 1 (SR1). NB, new bone (group 5); NNB, no new bone (group 1). **(B)** Photograph of an 8-week ossicle induced by PCL scaffolds coated with rhBMP-2, HA, Matrigel, and SR1 (group 5).

**FIG. 2.**
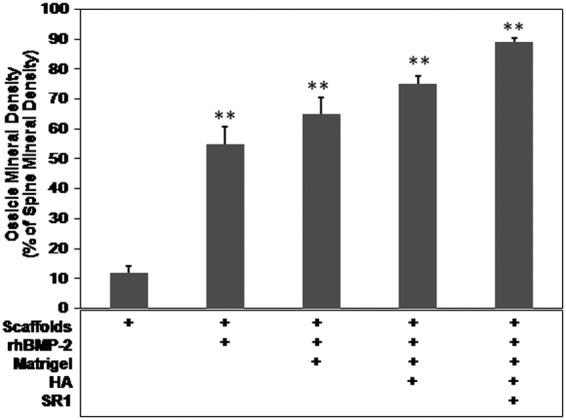
Comparison of mineral densities of ossicles among groups. The ratios of the average mineral density of 8-week *de novo* ossicles to spines of the same animals were calculated and expressed as a percentage of spine control (**p<0.01, compared with group 1). Data are presented as the mean±SE (*n*=6).

We introduced HA, Matrigel, and/or SR1, with or without rhBMP-2, to PCL scaffolds and implanted these biomodified scaffolds into mice. We evaluated the formation and mineral density of *de novo* ectopic ossicles by micro-CT, 8 weeks after implantation. Fig. 1A and B shows the representative *de novo* ossicle induced by PCL scaffolds coated with rhBMP-2 plus Matrigel, HA, and SR1 (groups 5). PCL scaffolds coated with vehicle (group 1; bare scaffolds, Fig. 1) or with Matrigel, HA, and/or SR1 without rhBMP-2 (groups 6–10) induced no *de novo* ossicle formation (comparable to those of abdominal tissues without PCL scaffold implantation; data not shown). The average densities of *de novo* ossicles were evaluated and compared with spines by analyzing six areas on each ossicle and spine in each animal. Better ossicle densities were obtained for groups 3, 4, and 5 than for group 2 (*p*<0.01) (Fig. 2), indicating that the inclusion of HA, Matrigel, and/or SR1, together with rhBMP-2, enhanced the *de novo* bone density over rhBMP-2 alone. These data confirmed that the subcutaneous implantation of rhBMP-2 containing biomodified PCL scaffolds could induce the formation of high-density *de novo* ectopic ossicles comparable to those of authentic bones, such as spine. We selected group 1 instead of groups 6–10 as a negative control (PCL scaffolds coated with vehicle without rhBMP-2) for further histological and hematopoietic characterization studies.

### Typical bone marrow architecture and multiple hematopoietic cells in *de novo* ectopic ossicles

**FIG. 3.**
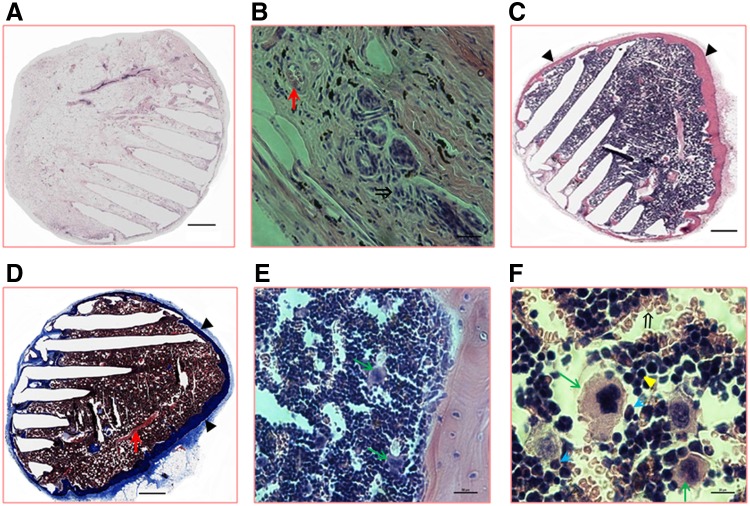
Architectures and cellular elements of 8-week nodules and ossicles. Representative views of *de novo* nodules or ossicles at 8 weeks post-PCL scaffold implantation. PCL scaffolds were coated with vehicle **(A, B**; group 1**)** or coated with rhBMP-2 plus HA, Matrigel, and SR1 **(C–F**; group 5**)**. Samples were stained with hematoxylin and eosin **(A–C, E, F)** and Trichrome **(D)**. **(A, B)** Showing *de novo* nodule architectures, blood vessels (↑), and proliferative mesenchymal cells (⇒). **(C, D)** Showing newly generated bones (▲). **(E, F)** Showing multilineages of hematopoietic cells, consisting of megakaryocytes (↑), monocytes (⇑), granulocytes (□), and erythrocytes (▲). Scale bars: 500 μm **(A, C, D)**, 50 μm **(B, E)**, and 20 μm **(F)**.

We examined the histological structures and the cellular components of the *de novo* ossicles using H&E and trichrome staining. The architectures of the 8-week *de novo* ossicles are shown in Fig. 3. The vehicle-coated PCL scaffolds (group 1) induced no *de novo* bone/bone marrow formation, only several blood vessels, along with mature red blood cells within these blood vessels, and proliferative mesenchymal fibroblastoid cells (Fig. 3A, B). Groups 2–5, and particularly group 5 (Fig. 3C, D), showed extensive remodeling of the *de novo* ossicles and the formation of typical marrow architecture. The 8-week-mature ossicles were filled with multilineages of hematopoietic cells, including monocytes, granulocytes, erythrocytes, and megakaryocytes, indicating the occurrence of hematopoiesis (Fig. 3E, F).

### Development of clonogenic hematopoietic precursors within mature ectopic bone marrow

**FIG. 4.**
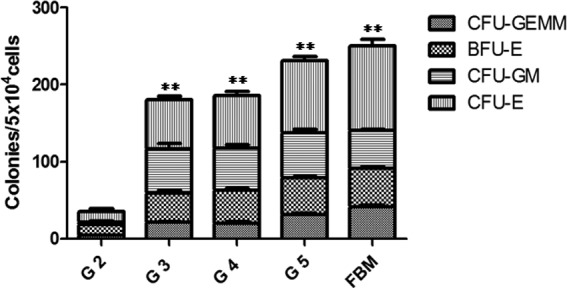
Hematopoietic progenitors and precursors in 8-week ossicles. The frequencies of total hematopoietic colony-forming units were significantly higher in ossicles developed from PCL scaffolds coated with rhBMP-2 plus HA, Matrigel, and/or SR1 (groups 3–5) than in ossicles derived from PCL scaffolds coated with rhBMP-2 alone (group 2) (**p<0.01). Data are presented as the mean±SE (*n*=6).

We evaluated the frequency of hematopoietic progenitors and precursors within the *de novo* generated ectopic bone marrow using CFU-assays. With the exception of group 1 (G1), all other groups (G2–G5) showed the presence of CFU-GEMM, BFU-E, CFU-GM, and CFU-E (Fig. 4). Group 5 showed the strongest response, with the total numbers of CFUs comparable to those found in the femoral marrow of the same animals (*p*>0.05). The frequencies of CFUs were lower in group 2 when compared with femoral marrow and groups 3, 4, and 5, which reflected the lack of Matrigel, HA, and SR1 coatings in group 2.

### HSCs reside in the *de novo* OM

**FIG. 5.**
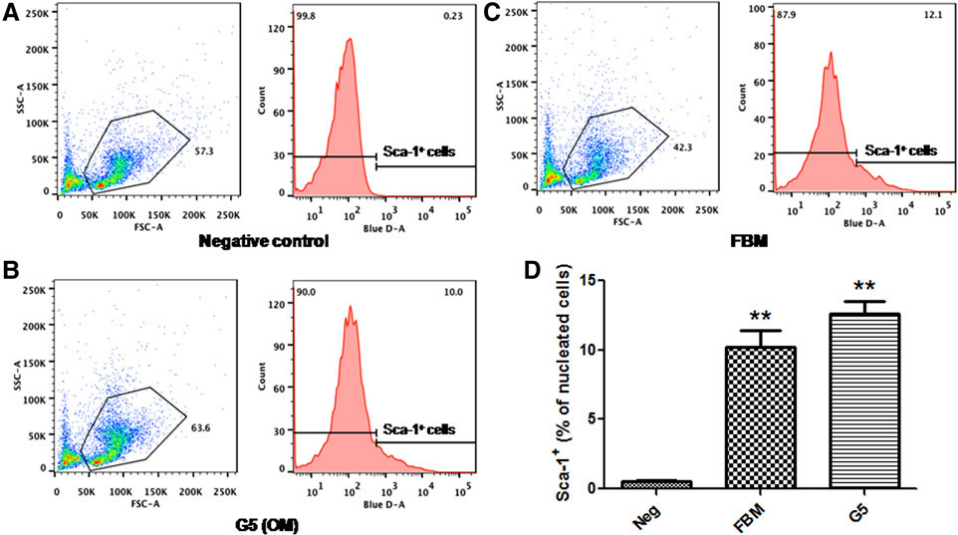
Hematopoietic Sca-1^+^ stem cells. **(A–C)** The percentages of Sca-1^+^ HSCs obtained from the negative control, *de novo* ossicle marrow (OM; G5: group 5) and femoral bone marrow (FBM), respectively. **(D)** Statistical analysis of Sca-1^+^ cells among three groups. Group 5 OM versus negative control: **p<0.01; group 5 OM versus FBM: *p*>0.05. Data are presented as the mean±SE (*n*=5).

The presence of multipotent or pluripotent stem cells in the *de novo* ossicles was determined by flow cytometry using a FITC-labeled anti-Sca-1 antibody. Stem cell antigen-1 (Sca-1) is the most common marker for murine HSCs and is used to identify the primitive murine HSC population.^24,25^ Sca-1^+^ HSCs are able to maintain the bone marrow stem cell pool throughout their lives, while the expression of Sca-1^+^ decreases upon differentiation into other mature cell types. Our results indicated a comparable percentage of Sca-1^+^ HSCs derived from the *de novo* OM to that found in the femoral bone marrow (FBM) of the same animal (Fig. 5) (group 5 vs. negative control: 12.6% vs. 0.23%, *p*<0.01; group 5 vs. FBM: 12.6%±0.94% vs. 10.2%±1.21%, *p*>0.05).

### Synergistic effects of rhBMP-2 and SR1 on the induction of ALP activity in cells undergoing osteogenic differentiation

**FIG. 6.**
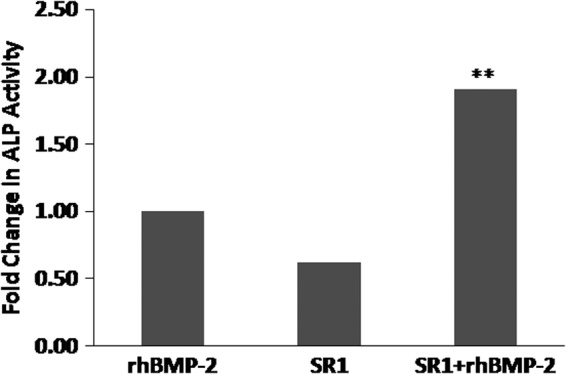
Synergistic effects of rhBMP-2 and SR1 on the induction of alkaline phosphatase (ALP) activity in ATDC5 cells. ALP activity was significantly higher in ATDC5 cells treated with 10 ng rhBMP-2 and 0.25 μM SR than in cells treated with 0.25 μM SR1 alone or 10 ng rhBMP-2 alone (**p<0.01, *n*=3).

We also examined the possibility that rhBMP-2 and SR1 stimulate the osteogenic differentiation in ATDC5 cells. The presence of 0.25 μM SR1 significantly enhanced the ALP activity in ATDC5 cells induced by 10 ng rhBMP-2 (Fig. 6). The combination index (>1) between 0.25 μM SR1 and 10 ng rhBMP-2 indicated synergistic effects of rhBMP-2 and SR1 on ALP or osteogenic differentiation activity in these cells. These *in vitro* synergistic effects on osteogenic differentiation in ATDC5 cells were consistent with their notable *in vivo* synergistic effects on the *de novo* bone-inducing activity, that is, histological examination of the *in vivo* bone formation at 8 weeks after implantation revealed better bone- and bone marrow-inducing activity with PCL scaffolds coated with rhBMP-2 plus SR1 than with PCL scaffolds coated with either SR1 (data not shown) or rhBMP-2 (Figs. 2 and 4).

## Discussion

The postnatal regeneration of functional and industrialized 3D organs in mammals has long been an important and challenging task. The goal of our study was to build a convenient and manageable 3D bone marrow model that would allow bioengineering, drug testing, stem cell expansion, and mechanistic studies of stem cell fate within a 3D microenvironment. Our initial finding in our earlier study was that bone marrow can be induced by *de novo* generation in adult rodents by subcutaneous implantation of rhBMP-2 coupled with DBMP. However, the DBMP is not an ideal bioscaffold as it does allow control of the shape and the thickness of the *de novo* generated ossicles. This disadvantage complicates subsequent reculturing or bioengineering of the resulting ossicles, as well as the maintenance of their *in vitro* stem cell and progenitor activities.

Our aim in the present study was to improve our previously established *de novo* bone marrow systems^15^ to develop a more efficient and convenient method for generating manageable 3D bone marrow. Therefore, we specifically changed our materials from DBMP to precast PCL scaffolds and we modified these PCL scaffolds with different biologics. PCL is an FDA-approved biodegradable polymer used for bone tissue engineering applications. However, PCL polymers alone have insufficient osteoinductivity to support bone/bone marrow growth.^19,26^ We therefore improved the osteoinductivity by using porous PCL scaffolds coupled with our comprehensive combinations of rhBMP-2 plus HA, Matrigel, and/or SR1, to develop new *de novo* generated ectopic 3D bone marrow models. We demonstrated that these new biomodified thin PCL scaffolds could promote sufficient and homogeneous *de novo* bone and bone marrow formation, as indicated by micro-CT (Figs. 1 and 2) and histological (Fig. 3) and hematopoietic characterization (Figs. 4 and 5) studies. The coating of these Matrigel-based biomaterials had no effect on the porosity of the scaffolds or the formation of trabecular bones after implantation into the animals (Fig. 3). These *de novo* ossicles contain multilineages of hematopoietic cells, including Sca-1^+^ HSCs (Figs. 3–5). Multipotent self-renewing Sca-1^+^ HSCs are capable of replenishing all blood cell lineages throughout their lives, but their development and maintenance depend on their location in a hematopoiesis-inducing microenvironment.

Bone morphogenetic proteins play an important role in bone regeneration^27,28^; rhBMP-2, in particular, can promote efficient bone formation.^29,30^ Accumulating evidence and our own previous studies indicate that subcutaneous implantation of bioscaffolds (e.g., DBMP) coupled with rhBMP-2 promote the differentiation of local mesenchymal cells and the subsequent support of HSC differentiation within the *de novo* regenerated bone marrow.^15,31^ We have used the same concept and principle in the present study to develop new ectopic *de novo* bone marrow through subcutaneous implantation of PCL scaffolds coated with Matrigel, HA, and/or SR1, in addition to rhBMP-2.

Matrigel is a gelatinous protein mixture isolated from Engelbreth-Holm-Swarm mouse sarcoma cells. Its major components are laminin, heparan sulfate proteoglycans, collagen IV, and entactin/nidogen^32^; these are all important components of the extracellular matrix (ECM) of the bone marrow microenvironment. Matrigel also contains transforming growth factor-beta, epidermal growth factor, fibroblast growth factor, and other growth factors.^33^ Matrigel promotes *in vitro* cell differentiation, but it also provides a critical 3D structure for stem cell culture that allows stem cell expansion without the need for feeder layers.^34,35^ We exploited these characteristics by introducing Matrigel into the precast thin PCL scaffolds as a way to mimic the *in vivo* ECM for controlled release of biologics and temporal regulation of cell proliferation and differentiation. The inclusion of Matrigel was critical for the development of hematopoiesis, because the scaffolds without a Matrigel coating showed lower CFUs within the induced ossicles (group 2) when compared with ossicles induced by scaffolds coated with Matrigel (groups 3–5). However, without the presence of rhBMP-2, Matrigel alone was unable to induce the formation of ectopic bone and bone marrow (data not shown). Matrigel has demonstrated a capability, both geometrically and mechanically, to sustain the rhBMP-2 activity in a similar manner to that seen with heparin.^36,37^ Three possible mechanisms can explain this action: prolonging the rhBM-2 half-life, decreasing interactions between BMP-2 and noggin (BMP-2 antagonist), and decreasing BMP-2 internalization.

HA is the major inorganic mineral within bone tissue. The HA-containing scaffolds were more bone mimetic for osteoinductivity, as these scaffolds (groups 4 and 5) showed a better ossicle density (Fig. 2) and greater hematopoietic activity (Figs. 3–5) within their associated bone marrow than did scaffolds without HA coating (data not shown).

SR1, a purine derivative, is an aryl hydrocarbon receptor antagonist. SR1 can cause 47-fold increases in *ex vivo* expansion of CD34^+^ cells and 17-fold increases in the number of engrafted CD34^+^ cells after grafting into immunodeficient mice.^38^ Our data demonstrated for the first time that the addition of SR1 increased the development of hematopoietic progenitors and precursors in ossicles (group 5) when compared with SR1-deficient groups (groups 2–4, Fig. 4). The synergistic effects of rhBMP-2 and SR1 on the *in vitro* ALP activity or osteogenic differentiation in ATDC5 cells (Fig. 6) were consistent with their notable synergistic effects on *de novo* induction of bone *in vivo* (Figs. 1–5).

## Conclusions

We developed a new and manageable 3D bone marrow model using a comprehensive combination of PCL scaffolds and biologics. The formation of mature ossicles was confirmed by micro-CT examination, which indicated a greater development of high-density bones when PCL scaffolds were biomodified with HA, Matrigel, and SR1 together with rhBMP-2 than with rhBMP-2 alone. These thin mineralized *de novo* bones contain functional hematopoietic bone marrow that resembles authentic bone marrow, as determined by structural, phenotypic, and clonogenic studies. This new model offers several advantages over the existing models as it is thin and easy to reproduce, evaluate, and subculture. It will provide an unusual opportunity for advanced bioengineering that caters to the different demands imposed by basic and clinical applications such as stem cell expansion, drug testing, and mechanistic studies of stem cell fate within a 3D HM.

## Abbreviations Used

3D: three dimensional
ALP: alkaline phosphatase
BSA: bovine serum albumin
CFU: colony-forming unit
DBMP: decalcified bone matrix powder
ECM: extracellular matrix
EDTA: ethylenediaminetetraacetic acid
FBM: femoral bone marrow
FBS: fetal bovine serum
HA: hydroxyapatite
H&E: hematoxylin and eosin
HM: hematopoietic microenvironment
IMDM: Iscove's Modified Dulbecco's Medium
OM: ossicle marrow
PBS: phosphate-buffered saline
PCL: polycaprolactone
rhBMP-2: recombinant human bone morphogenetic protein-2
Sca-1: stem cell antigen-1
SR1: StemRegenin 1
